# Evaluating the feasibility of implementing a prescription drug misuse prevention intervention in the community: a mixed methods study

**DOI:** 10.1186/s12889-023-15608-9

**Published:** 2023-04-21

**Authors:** Tamara Al Rawwad, Vaishnavi Tata, Matthew A. Wanat, Danielle Campbell, Douglas Thornton

**Affiliations:** 1 School of Social Work, The University of Texas Rio Grande Valley, 1201 W. University Drive, Edinburg, TX 78539 USA; 2 Department of Pharmaceutical Health Outcomes and Policy, University of Houston College of Pharmacy, 4849 Calhoun, Health 2, Houston, TX 77204-5000 USA; 3 Department of Pharmacy Practice and Translational Research, University of Houston College of Pharmacy, 4849 Calhoun, Health 2, Houston, USA

**Keywords:** Controlled substance prescriptions, Prevention intervention, Feasibility, Mixed-methods, Community-coalitions, Implementation

## Abstract

**Background:**

This study is part of a state-wide effort to promote the safe disposal of prescription medications and mitigate prescription drug misuse. The objective of this study was to evaluate the implementation of a two-component prevention intervention through Community Prevention Organizations (CPOs) in Texas. The first component involved the distribution of in-home disposal products (IHDP) and the second focused on providing education of the risks of prescription drug misuse.

**Methods:**

This study followed a mixed methods sequential explanatory study design. In the quantitative phase, the extent to which CPOs carried out the intervention was determined by the distribution rate – a proportion representing the number of IHDP distributed to end users from the amount of IHDP the CPO was shipped. This measure was used to organize the CPOs in to one of three performance categories. In the qualitative arm of the study, stratified random sampling was used to select five CPOs from each performance strata to participate in an in-depth, semi-structured interview about their distribution activity. The interview guide and the data analysis were guided by Bowen’s Feasibility Framework. The interviews were transcribed and analyzed using a content analysis approach by two research team members. All qualitative analyses were conducted in ATLAS.ti© V7.

**Results:**

There was a total of 47 CPOs contacted and asked to be part of this study. Of them, 44 CPOs participated in the quantitative phase of the study. This phase revealed that all CPOs had existing relationships with organizations throughout the community such as pharmacies and schools that could act as points of distribution. Following the quantitative phase, 15 CPOs were selected for more in-depth interviews about their distribution practices. In the qualitative phase, this finding was reinforced through the theme “partnerships with local institutions and ability to implement the intervention at community events”. Similarly, education promotion efforts were unanimously emphasized as a strategy to increase utilization of IHDP among end users. All CPOs indicated that the intervention was supplemental to their overall goals.

**Conclusion:**

CPOs have unparalleled access to community events, local institutions, and the general population they serve, thus, they have the potential to be active facilitators in implementing prevention interventions.

**Supplementary Information:**

The online version contains supplementary material available at 10.1186/s12889-023-15608-9.

## Introduction

Among initiatives to combat the ongoing opioid epidemic, the safe disposal of prescription drugs emerges as an effective way to prevent prescription medications, particularly pain medications, from being diverted or misused. According to the National Survey on Drug Use and Health (NSDUH), the most common source (50.8%) for the last pain reliever misused among people aged 12 or older was from a friend or relative in some way (i.e., being given them or taking them without asking) [1]. To mitigate the risk of unused or leftover medication being diverted or misused, safe drug storage and disposal practices are imperative.

While federal agencies have initiated medication take-back programs such as the Drug Enforcement Administration’s National Take-Back Day, the rate of proper disposal of unused medications in the United States remains relatively low [2]. The low rate of proposal medication disposal practices can be attributed to the burden available disposal options place on the end user. For example, the location of medication take-back boxes may be inaccessible to patients outside of certain hours or in a location out of their usual route. Depending on these factors, it is possible that end users simply find the burden of proper medication disposal not worth the benefit it provides. An alternative to having end users drive to the disposal site is to provide them with safe disposal options in the comfort of their own home. In-home disposal products (IHDP) allow end users the convenience of removing leftover or unused medications within their own home. Research shows that receiving IHDP for in-home disposal of unused opioids was associated with an increase in self-reported disposal and the likelihood of excess opioid disposal when dispensed in certain settings [3, 4]. In April 2022, the Food and Drug Administration posted a solicitation of public comments on the process of including IHDPs in the Opioid Analgesic Risk Evaluation and Mitigation Strategy (OA REMS) program that cited our prior work [5, 6].

At 47.2 prescriptions per every 100 individuals, the opioid prescribing rate in the state of Texas is close to the nationwide opioid prescribing rate [7]. In 2018, Texas reported a total of 1,402 opioid-involved overdose deaths [8]. Engaging Texans in proper disposal of opioid medications could greatly ameliorate the problem; however, as one of the most expansive states in the nation, Texas also exhibits a wide variety of communities. Texas is home to three of the ten largest cities in the United States, but it also has a significant number of rural communities. It is not viable to assume that the way a public health intervention is implemented in a metropolis will be successful in another community with a differing structural makeup. To successfully integrate IHDP throughout Texas communities, the cooperation of local community organizations is imperative. Understanding core aspects related to feasibility and sustainability is prudent with funding for this type of opioid-focused prevention becoming available through settlement funds across the US [9].

The Substance Abuse Prevention and Treatment Block Grant (SABG) is a program that provides funds to all 50 states, the District of Columbia, and other United States territories to support the planning, implementation, and evaluation of activities to ameliorate the issue of substance use [10]. At the time of this study, Texas had 47 SABG-funded Community Prevention Organizations (CPOs), each of which covered a mutually exclusive geographic area within the 11 public health regions in the state. CPOs consist of a wide variety of workers, from licensed community health workers to volunteers. Regardless of their demographic makeup, all CPOs play a vital role mobilizing the community to implement evidence-based prevention interventions and environmental strategies, in addition to monitoring, enforcing, or reporting at the grassroots level. Those that participated in this study all shared a common overarching goal of addressing Texas’s four prevention priorities: underage alcohol use, tobacco and nicotine products, marijuana and other cannabinoids, and prescription drug misuse. Their responsibilities also included utilizing the Strategic Prevention Framework (SPF) process: assessment, capacity building, planning, implementation, and evaluation, to guide the selection of target groups, implementation, and evaluation of the evidence-based, culturally appropriate, and sustainable prevention activities. In the state of Texas, CPOs attend one monthly meeting with the Texas Health and Human Services Commission (THHSC) to discuss changes, challenges, and successes. They receive federal and state trainings once a year that varies from year to year depending on the programs they are implementing. The state assigns a contact person that CPOs can communicate with to address any immediate concerns or needs.

In this study, Bowen’s framework was used because there is limited research on the implementation of this specific intervention in the community, and because the techniques that were used in the previous implementation were not guided by in-depth research [11]. The study will use the Bowen’s theoretical framework to examine the CPOs perception of factors that determine the feasibility and sustainability of this program in the community.

## Methods

### Study design

This study followed an explanatory sequential mixed methods design to evaluate the feasibility of the implementation of a prevention intervention by the SABG-funded organizations involved. Explanatory sequential designs are used when qualitative data collection is necessary to explain results from a quantitative data collection [12]. This design is particularly well-suited to situations where results from the quantitative arm of a study is used to group participants into different strata for further evaluation in the qualitative phase [12–14]. In this study, the qualitative arm of explanatory sequential design was used to explain the distribution rate of the CPOs presented in the quantitative data.

### Participants and recruitment

A list of SABG-funded organizations in Texas was provided by the Texas Health and Human Services Commission (THHSC) in March 2019. The research team identified the organizations that were still active for the coming fiscal year in May 2019. The list consisted of 47 organizations, covering 11 mutually exclusive geographic public health regions in the state of Texas. The research team reached out to all 47 organizations to explain the intervention and the involvement that would be required of them. Of the 47 organizations contacted, 44 chose to be involved in this intervention (*n* = 44), and the other 3 CPOs did not respond despite multiple contact trials. We learned later that these CPOs were no longer active. In the qualitative arm of this study, the 44 organizations were categorized into one of three different groups based on their IHDP distribution rate. Then, five organizations were randomly selected from each distribution strata to be included in the semi-structured interviews (*n* = 15). We used a stratified sampling approach to ensure proper representation of the sample’s subgroups. This also enabled us to obtain each subgroup’s input to and assess the difference in the factors affecting the feasibility of implementing the intervention among these subgroups. The number of the interviews was deemed appropriate because of the exploratory nature of this research homogeneity of the sample (they are all CPOs in Texas funded by the state). Additionally, Bertaux (p.35) suggests that the smallest acceptable qualitative sample size is 15 interviews [15]. Each of the 15 selected organizations nominated one employee who was directly involved in the implementation of the intervention to complete the interview. Informed consent was obtained from all 44 organizations prior to the beginning of the project. While all of the organizations were SABG-funded at the beginning of this study, there were changes in funding status during the time of the project. The research team began sending shipments of IHDP to the CPOs in July 2019, and distribution by the organizations to end-users began in September 2019. The study period evaluated was from September 2019 to February 2020. This study has been reviewed and approved by the [Blinded] Institutional Review Board.

### Intervention

A university for [redacted] State Opioid Response was contracted to perform the distribution, tracking, and the evaluation of the implementation of the intervention. The research team adopted the intervention components from SAMHSA’s strategies to prevent/reduce prescription drug abuse that focus on education and proper disposal of medications [16].

The intervention in this study consisted of two components: in-home disposal products (IHDP) and standardized educational material and science-based messaging about risks associated with sharing medications, safe storage of medications, and safe disposal of unused or expired medications in general, and prescription drugs in particular. IHDP are an at-home medication disposal method which comes in the form of a bag or an envelope, under two major categories: deactivation and incineration [17]. Deactivation products use a chemical process to denature medications added to the system, rendering them inactive, and Takeaway Medication Recovery System uses a mail-back approach for medication incineration. Although IHDP were distributed previously by the participant CPOs, there was no clear mechanism of tracking the utilization by the “end users” (the individuals who use these systems). In order to track the utilization of the disposal systems, a prepaid post card was attached to each bag/envelop that included a brief survey, in English and Spanish, about the type of medication disposed (prescription Vs nonprescription), the county/zip code, and number of pills/tablets. However, reporting End-user data is beyond the scope of this study. Both IHDP and the educational material were provided to the CPOs at no cost by the prescription drugs research center at a higher education institution using the funds from THHSC.

After identifying the CPOs that expressed an interest in participating in the delivery of the intervention, a Baseline Survey was sent to these CPOs. CPOs were not required to use specific means of intervention delivery or implementation. Some CPOs reported plans to deliver the intervention in local events at schools or at health fairs. Some reported plans to provide the intervention to a third party such as pharmacies and health clinics. And some CPOs reported plans to partner with different types of institutions they had established relationships with such as nursing homes and funeral homes. The participant CPOs did not receive monetary compensation for implementing the intervention.

### Data collection and measures

All 44 CPOs were requested to fill out an online survey entitled the *Baseline Survey*, in order to gather information on the populations the CPOs served, the programs they offered, the partnerships they had, and the amount of IHDP they believed they could distribute within the next six months. All CPOs were shipped the number of IHDP they estimated they could distribute in the six-month period based on the size of the population they serve, and their previous experience distributing IHDP for other projects. The estimated number of IHDP they requested indicated the demand.

The quantitative portion of this study used *Order Fulfillment Information* from the manufacturer to determine the number of IHDP each CPO was shipped. The Order Fulfillment Information refers to information provided by the manufacturers on how many IHDP were shipped to which CPOs. The number of IHDP each CPO was shipped was determined by their response to the Baseline Survey. To identify how many IHDP a CPO distributed, the CPO members were expected to fill out an online survey for each distribution event, referred to as the *Activity Tracking Form.* The form asked members of the CPOs, who are assigned by the CPOs to oversee the implementation of the intervention, to self-report the date and description of the distribution activity, the number of people in attendance, the number of IHDP distributed at this event, and which of the predefined Center for Substance Abuse Prevention (CSAP) strategies were employed at this event. Data from the Order Fulfillment Information and the Activity Tracking Forms were used to categorize each of the CPOs into one of three distribution strata. The high-level of distribution CPOs were defined as those that had distributed 75% or more of the IHDP they had been shipped. The medium level of distribution, distributed between 25 and 74% of the IHDP they had been shipped. Lastly, the low level of distribution CPOs reported distributing less than 25% of the IHDP they were shipped.

In the qualitative arm of this study, the point of interest shifted from the number of IHDP distributed to the facilitators and barriers/inhibitors of the acceptance, implementation, and integration of the intervention that influenced the distribution rate of the CPOs. Stratified random sampling was used to select five CPOs from each distribution rate strata to participant in semi-structured, in-depth interviews regarding their experience in implementing the intervention. Once the CPO was selected, the point of contact for that CPO, as reported in the Baseline Survey, was contacted to request an interview. The CPOs were asked to list the person more familiar with their group’s distribution activity as the point of contact. Therefore, the person(s) interviewed are assumed to be the most knowledgeable about the group’s intervention delivery. The first author of this paper, who has extensive experience in qualitative methodology and interviewing, developed the moderating questions for the interviews in accordance with Bowen’s framework for assessing feasibility, and conducted individual interviews with the selected coalitions’ leaders and staff. The questions asked within the interviews covered all the framework’s domains except for Limited-Efficacy Testing as it was beyond the scope of this study. The intention of each domain and examples of questions asked under each one can be found in the Supplementary Materials. The interviews were conducted using Zoom® teleconferencing software. Audio was recorded and subsequently transcribed using Rev^©^ services.

### Data analysis

Descriptive statistics were performed to report data from the Baseline Survey, Order Fulfillment Information, and the Activity Tracking Forms. Data collected from the Baseline Survey helped identify the characteristics of each CPO involved. From the Order Fulfillment Information, the research team was able to identify the number of IHDP each CPO had received. The Activity Tracking Forms provided the self-reported number of IHDP each CPO distributed.

In the qualitative phase, two members of the research team, who are trained in qualitative research, reviewed the transcripts, and cleaned up any discrepancies or issues within the recording and transcribing process. A content analysis approach, which focuses on summarizing the data elements in the data instead of creating theory or viewing the data in new ways [18], was used to analyze the data. Qualitative content analysis was an appropriate method because it matched the goals of the study which is to understand the intervention within the context of an established feasibility framework. After familiarization with the data by reading it multiple times, the two team members employed direct content analysis to code the data and created a codebook of themes based on the domains of feasibility mentioned previously. With the completion of the codes, the two team members underwent a second round of coding. In this stage, they separately coded the transcripts, specifically focusing on the facilitators and inhibitors’ themes that overlap under different domains. They then compared their codes lists. Interrater reliability was established by comparing the coders’ findings and the percentage of the agreement which was calculated by adding the number of times the two team members agreed on themes, then dividing that sum by the total number of data items. There was an 80% agreement between the two team members on the codes in the final list, which is at the 80% agreement needed for reliability in content analysis [19]. Disagreements were discussed between both researchers, which led to a 100% agreement on the selection of the final codes. Additionally, a fellow researcher who is trained in qualitative research conducted peer debriefing [20] of the data, to supports the credibility of the data and to provide a means toward the establishment of the overall trustworthiness of the findings.

## Results

### The baseline survey, order fulfillment information, and activity tracking forms

As seen in Table 1, the majority of CPOs reported that they have previously received IHDP to distribute within the areas they serve (*N* = 41, 93.18%). All participating CPOs reported offering services through schools (*N* = 44, 100%), and the majority reported providing services through community pharmacies (*N* = 31, 70.8%). Participants reported providing services through free clinics, nursing homes, physicians’ offices, hospitals, and emergency rooms, in addition to other institutions. Participants provided services to multiple age groups including children, teenagers, young adults, adults, and senior citizens. Almost one third (*N* = 16, 36.36%) self-identified as Youth Prevention Programs which provide evidence-based prevention activities before the onset of a substance use disorder [21]. Descriptive statistics regarding the amount of IHDP received by each CPO that was interviewed, the amount they distributed within their community, and their subsequent distribution strata can be found in Table 2.

**Table 1:** Sample characteristics (*N* = 44)

Variable	*N* = 44	%
Previously Received SUDS to Distribute		
Yes	41	93.18%
No	3	6.82%
Institutions and End-users that Received SUDS from Coalitions		
Hospitals	13	29.55%
Schools	44	100.00%
Physician Offices	14	31.82%
Free Clinics	18	40.91%
Emergency Rooms	6	13.64%
Nursing Homes	16	36.36%
Community Pharmacies	31	70.45%
Other	24	54.55%
Age Groups Served by Coalition		
Children (0–12)	19	43.18%
Teenagers (13–18)	37	84.09%
Young Adults (19–24)	44	100.00%
Adults (25–64)	34	77.27%
Senior Citizens (65 +)	31	70.45%
Youth Prevention Programs		
Yes	16	36.36%
No	28	63.64%
Programs Specific to Prescription Drug Misuse		
Yes	38	86.36%
No	6	13.64%

**Table 2:** CPOs included in semi-structured interviews

CPO Code	Number of SUDS Shipped	Number of SUDS Distributed	Percentage Distributed	Performance Stratum
1	9380	2225	23.72%	Low
2	1050	821	78.19%	High
3	3010	0	0.00%	Low
18	700	700	100.00%	High
19	1330	666	50.08%	Medium
20	7560	5349	70.75%	Medium
22	840	274	32.62%	Medium
26	2240	2040	91.07%	High
27	1540	0	0.00%	Low
28	420	0	0.00%	Low
34	5040	2000	39.68%	Medium
37&38^a^	7770	3329	42.84%	Medium
40	2800	2540	90.7%	High
42	1050	50	4.76%	Low
45^b^	630	1340	212.70%	High

^a^CPOs 37 and 38 were found to have been working together in their distribution efforts and were sharing SUDS. For this purpose, they will be reported together

^b^CPO 45 received additional SUDS from another CPO

At the end of the quantitative phase, 33 CPOs (75%) provided activity tracking forms which were used to determine the number of IHDP distributed by the CPOs. If a CPO did not complete any Activity Tracking Form, the research team contacted them to explore the underlying reasons and make sure that the CPO did not distribute IHDP. Upon confirming that CPOs had not distributed any IHDP, they were included in the low level of distribution stratum. One CPO reported distributing more than a 100% of the amount they were shipped. This was due to the CPO receiving additional IHDP from another CPO that was dissolved.

Table 2 shows the shipping and distribution information for all 15 CPOs that were interviewed.

This study’s research question: What do CPOs believe makes implementing the IHDP intervention feasible and sustainable in the community? was examined through the acceptability, demand, implementation, practicality, adaptation, integration, and expansion of the intervention, using Bowen’s framework of feasibility discussed above.

### Demand

Since this study examined the feasibility of the implementation and the sustainability of the intervention, and it was not intended to evaluate the impact of the intervention, the demand was estimated by the number of shipped IHDP based on the number that was requested by the CPOs. This number was estimated by the CPOs based on their previous experience with distributing IHDP for other projects, and their knowledge of the population and the areas they serve. Table 2 shows the number of shipped IHDP to each CPO.

### Semi-structured in-depth interviews

Seven themes emerged from the data that examined the study’s research question: what do CPOs believe makes implementing the intervention feasible and sustainable in the community?

- Theme #1: The intervention is desirable
- Theme #2: The intervention is needed
- Theme #3: CPO are creative with their Efforts
- Theme #4: The intervention should be seen as cost-effective
- Theme #5: Addressing structural Factors that Inhibit Adoption of IHDP
- Theme #6: Other organizations’ impact
- Theme #7: CPOs interest to continue addressing implementation challenges.

While all the themes will be discussed in this paper, please refer to Table I in the Supplementary Materials for the full list of examples of quotes for each code. In addition, to protect the anonymity of participants, actual names were replaced with the CPO’s number.

### Theme #1: The intervention is desirable

All participants stated that the prescription drug misuse prevention is a priority for their organizations, and the intervention is in concordance with their mission statements which made it acceptable and eased its integration into their existing programs. Additionally, the CPOs’ staff engagement with the intervention were other factors that increased the acceptance of the intervention among the staff. Participants frequently reported the preserved privacy and the autonomy of the end user over their medications as reasons why it was well-received by the populations they serve specifically women, senior citizens, and schools. Participants also highlighted the IHDP design being user friendly and instructions being easy to understand as reasons for its practicality.

“It's a supplemental that the state asks us to do, but it fits in because we are trying to reduce opioid overdoses and access to prescription drugs by youth” (CPO 37)

*“But at the end of the day, like I said, a lot of people are still fearful of going into the police department because of their immigrant status, or because lack of transportation. If we're able to actually go into the community and disperse these, they're easy to use” (CPO 42)*

*“People say, ‘What? It's just that?’ It's so simple and self-explanatory that people don't even believe that it's that simple” (CPO 34)*

Furthermore, all participants mentioned increased awareness of the intervention as a result of the CPOs’ efforts, end user engagement with the intervention, and the engagement from community partnerships as other contributors to the acceptability of the intervention.

*“Another positive thing is, that people are actually taking an account the severity of the opioid crisis, they're taking the initiative into great strides and saying, "We got to do something. Let's be proactive." They actually learn and they're wanting to know more information” (CPO 20)*

### Theme #2: The intervention is needed

All CPOs (*N* = 15) in the interviews indicated the need for intervention by their CPO and the communities they serve. Across all three level of distribution strata, the prevalence of medication hoarding, increased prescribing of prescription drugs, prevalence of prescription drug misuse in the communities the CPOs served, and limited access to other disposal methods, were cited as reasons for the need for the intervention. Additionally, IHDP were described as a disposal option that is available throughout the year and does not require transportation to a predetermined location.

“At one point about a year ago, there were 107 opioid prescriptions per every 100 residents. What that means is that there are people out in my community that have multiple prescriptions, two, three, or even more than that” (CPO 02)

“Our suicide rate is also very high, which goes directly with misusing the prescription drugs and opioids” (CPO 19)

### Theme #3: CPO are creative with their efforts

Partnerships with local institutions and community liaisons was the most cited facilitator for the implementation of the intervention across CPOs from all levels of distribution. Participants also cited the ability of CPO members to attended community events and having the flexibility to distribute IHDP and implement the intervention in a variety of local community venues as an important facilitator of the implementation.

*“We really focus a lot of our efforts on building capacity. What that means for us in real time is having these really solid working relationships with organizations in our community” (CPO 02)*

Participants reported different strategies that they allowed them to minimize their own resource burden while still maximizing the reach and utilization of the intervention. These strategies included bulk distribution of IHDP, collaboration with other similarly oriented groups, and being strategic in their selection of partnerships and avenues of implementing the intervention.

*“So, I was able to give them several hundred pouches that they then distributed straight back into our community, which I think was really effective” (CPO 02)*

In the interviews, respondents discussed various efforts they initiated to improve the implementation process. These efforts centered mainly on the creation of resources to encourage the adoption of the intervention among the end users, and the efforts at CPO’s part to increase educational promotion/trainings regarding the intervention to the community.

*“We had built a website called [Redacted] that we could point people towards” (CPO 18)*

*“We push it on our Facebook page as well, so we have a Facebook for the coalition” (CPO 38)*

Participants reported a variety of modifications and changes that they made to the intervention including adapting methods to increase the reach of the intervention, making modifications to the IHDP, and tailoring messaging and education efforts to fit the audience.

*“Another comment I wanted to add was that I believe one of our staff members had to type up some of this information in Spanish because we live in a predominantly Hispanic area where a lot of people do not speak English” (CPO 42)*

*“[Our service area] has a lot of Hispanics, we did add a sticker that had Spanish instructions on it. Now the postcard itself has Spanish on it but we added a sticker like a mail sticker, it's not very big we added it on, just stuck on the back to each one” (CPO 03)*

### Theme #4: The intervention cost-related factor

All participants cited cost related factors as significant determinants of the intervention adoption and integration by their CPO. Cost was also a determining factor for third parties the CPOs collaborated with and the end users of the IHDP. Based on their experience working with the populations and community partners, participants believed that it would be acceptable if there was a minimal cost associated with IHDP. However, they did also indicate that if the cost were placed onto the end user or the third party, then the rate of adoption may decrease. When asked to further elaborate, it was elicited that end users had other disposal methods, ones marketed by the Food and Drug Administration (FDA) and Environmental Protection Agency (EPA), with no cost associated.

*“I think, because if somebody is going to pay to dispose of meds, I mean if I were to look at it from my perspective, it would be easier for me just to throw them in the trash versus paying X amount of money for pouch to dispose the meds properly” (CPO 01)*

### Theme #5: addressing structural/process factors that inhibit adoption of IHDP

Interviewed CPOs reported several factors that inhibited the adoption of the intervention by their CPO or the populations they serve. Participants reported service area related factors including rurality, and language barriers.

*“That it doesn't have it translated in Spanish on the back. I think that maybe people may take them, and then be like, "I really don't even understand this” (CPO 42)*

Participants also reported lack of community awareness regarding the risks of prescription drugs coupled with lack of community awareness regarding IHDP posed the need for active marking to increase the awareness of the availability of the IHDP and educate on the risks of prescription drugs. Additionally, some participants mentioned that younger population being not as engaged with the intervention as a challenge they faced. A few participants mentioned that end user being uncomfortable with disclosing personal information related to what type of medications they are taking and be hesitant to fill out the postcard attached to the IHDP for any reason as a challenge.

*“Most people don't know that it's that big of a problem. It seems like okay; it might be a problem in New York or somewhere else, but it's not a problem here. Opioid use is not a problem in our community. I think that's something we're seeing in our events is they don't really see it as a problem. They know it could be used, to be a problem, but I don't think they really understand it in our community” (CPO 34)*

Several participants cited factors related to the area their CPO serves that made it difficult to accept or implement the intervention. These reasons included rurality and language barriers. Other inhibitors for the acceptance of the intervention discussed were limitations on structural capacity. Some CPOs reported not having the infrastructure necessary to house the volume of IHDP or the personnel to implement the intervention.

*“Definitely one of the factors for us is distance of all our locations. […] County is a large county. It's very divided” (CPO 26)*

*“As it is here, there's the language barrier. For [the vender] to be able to provide leaflet information both in English and Spanish, it's a good thing because you target a whole bigger population, demographic area” (CPO 20)*

Participants from all distribution level reported having difficulties in tracking the implementation of the intervention and their IHDP distribution efforts. An additional factor that was cited exclusively by CPOs with low distribution rate and contributed to the challenges they faced when implementing the intervention was organizational mishaps. These mishaps included missing opportunities to implement the intervention, miscommunication, or a lack of communications between staff, and not documenting their distribution efforts.

When inquiring about other challenges the CPOs faced, the majority of CPOs with low distribution rate cited changes in state funding as a factor that interfered in their ability to adopt the intervention and implement it.

*“Now that CPO it's kind of sad. It's kind of faded away because there's not a lot of involvement since there wasn't a funding source to it. And so, our organization took it upon herself because we are in prevention education, and so a lot of the contacts I have, I'm able to disperse them. So, the shipment came in like beginning of September, but my contract ended August 31*^*st*^*” (CPO 01)*

### Theme #6: Other organizations’ impact

Respondents did cite some external factors that made the implementation easier, specifically in regard to sharing of monetary burden of the intervention with other entities and partners.

*“My organization could say, "Hey, your CPO wants to do this, we could purchase the pouches and we can partner, and you guys can do the manpower and work on a Saturday" (CPO 01)*

Some of the challenges reported by CPOs with high and moderate distribution rate interference from outside groups/overlapping services with the services the CPO provides, and the CPO coverage of counties not catered to by any other funded organizations.

### Theme #7: CPOs interest to continue addressing implementation challenges

Participants reported targeting other areas or populations and expanding partnerships with community entities and liaisons, as well as improving structural capacity, and increasing the focus on IHDP distribution and making it one of the main activities. CPOs with low and moderate distribution rate reported plans for finding alternative means of IHDP distribution and plans to develop a distribution tracking system to document and monitor the implementation of the intervention.

When asked about the perceived positive and negative effects that expanding the intervention could have on their CPO, participants stated numerous positive effects such as expanding the reach of their prevention efforts, building the CPO’s viability in the community, targeting multiple sectors interested in prescription drug misuse prevention at once, and most importantly, providing the communities they serve with the necessary resource to mitigate prescription drug misuse and abuse. On the other hand, consequences, or negative effects such as cost associated with the expansion and interfering with other activities and programs that are run by the CPO were also reported.

*“It would give us a chance to partner with more organizations in our community, to interact with more community members, educating them while creating awareness... Yeah, more positives than anything else” (CPO 26)*

*“I think it could inhibit some of the progress we're making on some of our partnerships” (CPO 28)*

## Discussion

In this study, Bowen’s framework [11] was used to assess the feasibility of providing an intervention that targets the three levels of health promotion: awareness, education, and behavior change, through CPOs across the state of Texas. Results indicate that What do CPOs believe makes implementing the intervention feasible and sustainable in the community is that there is a demand for the intervention among both the CPOs and the populations they serve, the, intervention is desirable, CPO were creative with their efforts, the intervention cost-related factors, addressing structural factors that inhibit adoption of IHDP, other organizations’ impact, and CPOs’ interest to continue addressing implementation challenges. Although these factors might not be unique to this intervention, however, this is one of the first studies that examines the feasibility of implementing this specific prescription drug misuse intervention in the community, through CPOs.

Although data shows that Texas is about average with respect to opioid prescribing, during the 20-year period from 2000 to 2019, many CPO members reported being concerned over the prevalence of prescription drug misuse within the communities they serve attributing the prevalence to the increased prescribing of controlled substances, and obtaining prescription medications from different resources [22]. Medication hoarding stemming from cultural practices, a lack of awareness regarding the availability of safe drug disposal options, or limited transportation to locations where safe drug disposal is provided was also seen as a potential problem that could exacerbate the issue of prescription drug misuse, thereby generating the demand for IHDP among the CPOs due its uniqueness. Increasing the awareness of the availability of the intervention among end-users through active marketing by CPOs to generate the demand is a technique that has been used in other prevention interventions [23].

A crucial component to the successful implementation of this intervention was the partnerships the CPOs developed with local institutions and community liaisons such as pharmacies, student organizations, clinics, and schools in addition to collaborating with other similarly oriented groups. The latter indicates that funding agencies should encourage collaboration between organizations and other entities as part of the awards granted [24]. Data suggested that factors that facilitated the acceptability of the intervention also influenced the practicality and integration of the intervention into the CPOs’ existing programs and activities. These results indicate the importance of considering some factors when choosing an intervention such as the organization’s available resources, the intervention’s suitability and fit with the goals of the organization, and to what extent it would fulfill the needs of both the organization and the end-user, to guarantee its sustainability, especially when there is a cost attached to intervention.

Some areas that challenged the feasibility of providing this intervention through the CPOs such as the general lack of awareness by some of the CPOs communities regarding prescription drug misuse, posed a challenge when explaining the need for intervention, due to the rural nature of the communities the CPOs serve, their cultural beliefs and practices, and language barriers. In order to lessen the lack of awareness, participants emphasized the importance of the educational component of the intervention, and its potential effect on increasing end users’ acceptance and intention to use the IHDP. Adding stigma reduction efforts, through culturally appropriate and tailored messages, and including trusted community influencers such as faith leaders after providing training for them, would help in promoting the behavior change and increasing the acceptance of the intervention. Faith leaders are able to influence health behavior on the individual, socio-cultural, and environmental levels [25]. Adding Spanish translation to the materials to reach some of the predominantly Latinx communities who have limited English proficiency, as suggested by CPOs, emphasized once more, the need to have community stakeholders, external to the research team, providing input during the planning and throughout the implementation phases, and including representatives from various groups to help foster intervention success [26].

Since almost all participant CPOs were federally or state funded, loss of state funding impacted the implementation of the intervention in some CPOs, and it explained why some fell under the low distribution rate category. Loss of state funding caused some coalitions to disintegrate, which consequently affected their ability to retain personnel who were responsible for implementing the intervention or secure a physical space to house the volumes of the IHDP. This indicates the need for continuous governmental support for these CPOs in order for them to continue their important and unique role in advancing prevention efforts in the community. It also indicates the importance of encouraging collaboration among CPOs and between CPOs and other agencies to compliment and sustain their efforts [24].

One factor that was unique to the CPOs with low distribution rate that challenged the implementation of the intervention was organizational mishaps. This included not receiving the intervention in time to be implemented at the scheduled activities, not organizing the implementation activities in a timely manner, and lack of internal communication between predecessor and successor personnel. This highlights the importance of internal communication and the importance of regular communication between CPOs and the institution that created the intervention to avoid and be aware of such mishaps.

To enhance the probability of adoption an intervention usually must be associated with minimal effort and psychological, social, or monetary response costs. Although CPOs consider IHDP cost-effective, and that the intervention was provided at no cost to the CPOs, involved third parties, and the end users, participants identified potential issues with others adopting the intervention based on cost. This indicated the importance of considering the cost associated with an intervention, including its price and the cost of its delivery in the early stages of program planning. Participants provided some solutions to resolve this potential challenge such as sharing monetary burden with other organizations and seeking funds from multiple sources to cover the cost and support the continuation of the intervention. However, besides minimizing costs, there should be reinforcement for the initiation and maintenance of behavior change. This can be done by making the product itself—the positive health behavior—reinforcing. A more sustainable way is for CPOs to encourage prescribers and pharmacies to provide the intervention as part of the patient education and drug misuse prevention efforts that can be reimbursed through insurance, a policy that can be suggested to be included in the Substance Use-Disorder Prevention that Promotes Opioid Recovery and Treatment for Patients and Communities Act [27].

While all participants reported difficulty in tracking the distribution of IHDP, only few CPOs with low distribution rate mentioned intentions to develop tracking systems to appropriately evaluate the impact of the intervention. This emphasizes the need to increase the CPOs’ awareness of the available methods of tracking the IHDP, and creating new means of distribution that would allow effective tracking, such as a centralized log where all CPOs enter their distribution, and assigning specific individuals to follow up with third parties or end-users.

Despite all the challenges reported by the CPOs, participants were proactive and reported effective strategies to overcome these challenges. CPOs were actively creating resources and adapting and modifying the intervention to expand its reach. They were also wisely evaluating the expansion plans by assessing positive and negative effects of the expansion. Their plans were informed by their own experience and knowledge of the needs of the populations they serve including reaching other areas and populations and expanding partnership with community entities and liaisons.

This study suggests that a community-based distribution and education approach to mitigating prescription drug misuse through IHDP is imperative. CPOs have the potential to effectively meet the unique needs of their communities. However, in order to successfully integrate the intervention in communities through CPOs throughout Texas, there must be some central regulatory body that keeps track of shipping and distribution efforts as well as gives the CPOs benchmarks to meet in their efforts. Additionally, it is crucial that CPOs target the distribution efforts and provide the IHDP to the end-users’ groups and entities that would be more likely to use them, otherwise, the intervention will not be cost effective. Examples of these groups would be patients who are prescribed opioids and their families, pharmacies, and nursing homes.

### Limitations

Limitations in this study includes the fluctuations within funding status of CPOs, where some CPOs lost their federal fund and caused them to stop their work during the study period. These changes also caused other CPOs to absorb the shipments of unfunded CPOs in some cases, making the research team’s tracking efforts more difficult. The major research limitation is the reliance on self-report, and the bias that due to the social stimulus characteristics of the situation. Additionally, participants in the qualitative phase were asked to recall information over a certain period of time, so this can lead to recall bias due to participants not remembering accurately or omitting details. Another limitation is related to the nature of qualitative research, that findings of this study cannot be generalized and extended to wider populations. Despite these limitations, this study describes the first evaluation of a state-wide implementation of in-home disposal products. Understanding how IHDP can be utilized effectively is of critical need with communities receiving opioid litigation settlement funds.

## Conclusions

This study systematically examined the facets of feasibility and the potential programmatic elements to be included in future iterations of the intervention to be implemented in different settings and among different populations. The results provided a detailed view of the intervention and illustrated a demand for it, and it can help researchers identify the mechanisms that may facilitate the adoption and the implementation of a prevention intervention through community organizations. In addition, this study added value of capturing the voices of CPO leaders who are in the field, in a research study, and highlighted that the CPOs’ commitment to and engagement with the intervention were key to its successful implementation. The findings of this study are likely to help researchers and practitioners better understand the nuances of implementing interventions in various communities and addressing the challenges and potential solution for these challenges. Further research could assess if the intervention will work across diverse populations and settings in comparison to other alternatives.

## Supplementary Information

**Additional file 1.** Interview Moderating Guide.

**Additional file 2.** Feasibility Manuscript Final Coodebook.

## Data Availability

Data will be available upon request.

## Abbreviations

CPO: Community prevention organizations
CSAP: Center for substance abuse prevention
EPA: Environmental protection agency
FDA: Food and drug administration
HHS: United States department of health and human services
IHDP: In-home disposal products
SABG: Substance abuse prevention and treatment block grant
SMF: Social marketing framework
SPF: Strategic prevention framework
THHSC: Texas health and human services commission

## References

[1] Substance Abuse and Mental Health Services Administration, Key substance use and mental health indicators in the United States: Results from the 2018 National Survey on Drug Use and Health. Rockville: Department of Health and Human Services; 2019. Retrieved from https://www.samhsa.gov/data/sites/default/files/reports/rpt29393/2019NSDUHFFRPDFWHTML/2019NSDUHFFR090120.htm.

[2] Egan KL (2017). From dispensed to disposed: evaluating the effectiveness of disposal programs through a comparison with prescription drug monitoring program data. Am J Drug Alcohol Abuse.

[3] Brummett CM (2019). Effect of an activated charcoal bag on disposal of unused opioids after an outpatient surgical procedure: a randomized clinical trial. JAMA Surg.

[4] Lawrence AE (2019). Effect of drug disposal bag provision on proper disposal of unused opioids by families of pediatric surgical patients: a randomized clinical trial. JAMA Pediatr.

[5] Imarhia F (2020). Prescription drug disposal: products available for home use. J Am Pharm Assoc (2003).

[6] Food and Drug Administration Providing Mail-Back Envelopes and Education on Safe Disposal With Opioid Analgesics Dispensed in an Outpatient Setting; Establishment of a Public Docket; Request for Comments. 2022; Available from: https://www.federalregister.gov/documents/2022/04/21/2022-08372/providing-mail-back-envelopes-and-education-on-safe-disposal-with-opioid-analgesics-dispensed-in-an. [Cited 2022].

[7] Centers for Disease Control and Prevention (CDC). U.S. Opioid Prescribing Rate Maps. 2017 3 October 2018; Available from: cdc.gov/drugoverdose/maps/rxrate-maps.html. [Cited 5 Oct 2019].

[8] National Institute on Drug Abuse. Opioid-Related Overdose Deaths. 2018 February 2018; Available from: https://www.drugabuse.gov/drugs-abuse/opioids/opioid-summaries-by-state/west-virginia-opioid-summary. [cited 5 Oct 2018].

[9] National Academy for State Health Policy. State Approaches for Distribution of National Opioid Settlement Funding. 2022 [cited 2023; Available from: https://nashp.org/state-approaches-for-distribution-of-national-opioid-settlement-funding/.

[10] Substance Abuse and Mental Health Services Administration (SAMHSA). Substance Abuse Prevention and Treatment Block Grant. 2020 April 12 2020; Available from: https://www.samhsa.gov/grants/block-grants/sabg. [Cited 13 Aug 2020]. 27054226

[11] Bowen DJ (2009). How we design feasibility studies. Am J Prev Med.

[12] Creswell JW, Clark VLP (2017). Designing and conducting mixed methods research.

[13] Morgan DL. Integrating qualitative and quantitative methods: A pragmatic approach. Sage Publications; 2013.

[14] Abbas M, Tashakkori CBT (1998). Mixed methodology: combining qualitative and quantitative approaches (Applied Social Research Methods) applied social research methods.

[15] Modell J (1982). Biography and society: the life history approach in the social sciences. The Oral History Rev.

[16] Administration, S.A.a.M.H.S., Opioid Overdose Prevention TOOLKIT. 2019.

[17] Imarhia F (2020). Prescription drug disposal: Products available for home use. J Am Pharm Assoc.

[18] Drisko JW, Maschi T (2016). Content analysis.

[19] Bakshi P (2018). Development and validation of an HPLC-UV method for analysis of methylphenidate hydrochloride and loxapine succinate in an activated carbon disposal system. J Pharm Anal.

[20] Lincoln YS, Guba EG (1985). Naturalist inquiry.

[21] Commission, T.H.H.S. Youth Substance Use Prevention Programs. 2020; Available from: https://hhs.texas.gov/services/mental-health-substance-use/youth-substance-use/youth-substance-use-prevention-programs. [Cited 2 Dec 2020].

[22] Casner PR, Guerra LG (1992). Purchasing prescription medication in Mexico without a prescription. The experience at the border. West J Med.

[23] The One-Stop Clearinghouse for Global Prep Resources. 2022; Available from: https://www.prepwatch.org/.

[24] Robertson EB, Sims BE, Reider EE (2012). Partnerships in drug abuse prevention services research: perspectives from the national institute on drug abuse. Adm Policy Ment Health Ment Health Serv Res.

[25] Heward-Mills NL (2018). The role of faith leaders in influencing health behaviour: a qualitative exploration on the views of Black African Christians in Leeds, United Kingdom. Pan Afr Med J.

[26] Klesges LM (2005). Beginning with the application in mind: designing and planning health behavior change interventions to enhance dissemination. Ann Behav Med.

[27] Congress U. Substance Use–Disorder Prevention that Promotes Opioid Recovery and Treatment for Patients and Communities Act. In 115th Congress. 2018. Retrieved from https://www.congress.gov/bill/115th-congress/house-bill/6.

