# Supplementary material for: Evaluating the feasibility of implementing a prescription drug misuse prevention intervention in the community: a mixed methods study
Source: BMC Public Health. 2023 Apr 21;23:728. doi: 10.1186/s12889-023-15608-9 (PMC10120215; doi:10.1186/s12889-023-15608-9)
Supplement: Supplementary file 1 — Additional file 1. Interview Moderating Guide. [file 12889_2023_15608_MOESM1_ESM.docx]

**Additional File (1): Interview Moderating Guide**

| **Interview guide based on domains within Bowen’s framework for assessing feasibility** | |
| --- | --- |
| **Demand** | *Estimated or actual use of intervention activities in a defined target group* |
|  | To what extend are IHDP currently used in your CPO? |
|  | How do you perceive the demand of IHDP in your CPO? |
|  | What are some factors that would affect the demand of IHDP at your CPO? |
|  | How many people used IHDP? |
|  | What were their demographic characteristics? |
| **Acceptability** | *How the participants and the CPOS react to the intervention* |
|  | To what extent is IHDP are suitable to be distributed by your CPO? |
|  | What makes it suitable for your CPO? |
|  | Probing question: Is it size, easy to use, etc. |
|  | What makes it suitable for the population you serve? |
|  | What makes it suitable for the locations you serve? |
|  | What are some challenges your CPO faced regarding the acceptability of IHDP by the staff? |
| **Implementation** | *The extent, likelihood, and manner in which an intervention can be fully*  *implemented as planned and proposed* |
|  | What are the resources needed and provided by CPO to implement the IHDP distribution? |
|  | What type of activities do you perform to distribute the IHDP? |
|  | Probing question: is it handouts, training, presentations, etc.? |
|  | Would you say the distribution of IHDP was implemented as planned? Please elaborate |
| **Practicality** | The extent to which an intervention can be delivered when resources, time,  commitment, or a combination of these are constrained in some way |
|  | What are the factors that made the distribution of IHDP easy? |
|  | What factors that made the distribution of IHDP difficult? |
|  | How do you describe the efficiency, speed, or quality of implementation of IHDP by your CPO? |
|  | What efforts has your CPO made to make the distribution more efficient? Give examples: (Incorporate the distribution with other interventions, use educational materials, etc.) |
|  | What were some of the positive effects on the participants? |
|  | What were some of the negative effects on the participants? |
|  | In your opinion, is the cost of the IHDP reasonable in comparison to its value? How so? |
| **Adaptation** | *The extent changes made to the IHDP distribution means to develop a new format or to be used it in a different population* |
|  | 1. Has your CPO modified their activities in regard to the distribution of IHDP? |
|  | 2. Has your CPO developed new activities to achieve outcomes related to the distribution of IHDP? If yes, how so? |
|  | 3. Has your CPO compared the outcomes of distributing IHDP among two different groups or population? If yes, can you explain the process? |
| **Integration** | *The observed system/institutional changes that happened due to the IHDP integration* |
|  | To what extent does the distribution of IHDP fits with your CPO infrastructure and resources? |
|  | To what extent can IHDP distribution be sustainable by your CPO? |
|  | What would cost your CPO to integrate IHDP as part of its continuous programs? |
|  | What changes would be made to your CPO’s infrastructure/resources so IHDP can be integrated as part of your CPO’s programs? |
| **Expansion** | *The extent to which the IHDP distribution can be expanded* |
|  | What is the estimated cost on your CPO if you were to expand the IHDP distribution efforts? |
|  | How well does the distribution of IHDP fit with CPO’s goals and culture? |
|  | What negative effects would the expansion of IHDP distribution have on your CPO? |
|  | What positive effects would the expansion of IHDP distribution have on your CPO? |
|  | What are the potential effects or disruption to your CPO’s activities due to the expansion of IHDP distribution efforts? |

Table I: Themes, codes, and corresponding example quotes

| Theme #1: The intervention is desirable | Aligns with Coalition CPO Mission Goals | “Prescription drugs is one of the priority areas that we address. The distribution of medication disposal pouches is one of our activities and overall objectives” (CPO 26)  “We have three basic pieces to our strategic plan. One of them is the reduction of prescription drug abuse among students and families. We look to do that through a myriad of ways. One is disposable options. The disposable pouch fits in that piece of our strategic plan, and it's coupled with permanent drop boxes, and the medication take backs we do twice a year. That is one piece of that section of our strategic plan” (CPO 28)  “I mean, they're suitable because they're exactly what we... we're in the prevention business. We want to prevent the misuse of prescription drugs and this is exactly what... is a tool, a tangible tool that someone can go home and clean up their medicine cabinet and it's something that they can do. So it fits right into our mission, it allows us to provide them information either at a health fair or a presentation or some other avenue, but then also a take away, something in their hand that they can take away. So for that, it's very suitable” (CPO 03)  “It does fit very well in the population that we serve, and then basically on what we do as far as our prevention efforts out in the community” (CPO 20) |
| --- | --- | --- |
|  | Prior Experience with Intervention | “For the last few years, we actually had been using the [..] pouches prior to the donation that we had received. We had received a grant from the state a couple years back, and we were able to order a significant amount” (CPO 42)  “Actually, one of the pharmacies that we work with, because before when we had the grant ourselves, we were able to purchase the pouches. That time, we purchased on of those big containers” (CPO 34) |
|  | IHDP Being Supplemental/adjunct | “use as a starter for a conversation with the community” (CPO 26)  “And that's where we were able to disperse pouches on an individual basis. We're to, "Hey, I need you to do anger management presentation to parents at a local counseling center," couches with you because, "Hey, this is an easy way to dispose pouches even though the presentation may have not been about opioid use, but the resource that you can have if you are interested"” (CPO 01)  “It's a supplemental that the state asks us to do, but it fits in because we are trying to reduce opioid overdoses and access to prescription drugs by youth” (CPO 37)  “It's just add on to what we're already out there for services” (CPO 45) |
|  | Appealing to Schools | “And actually I did get a couple of phone calls later from a counselor who had been by one of our school resource booth and said, "Hey, can you bring me 10 of those for our nurse” (CPO 03) |
|  | Appealing to Senior Citizens | “But I've seen more of a proactive stance within the older population because they do understand the importance of not being able to hoard, if I may, they're medications that they're not currently using because of the risk of, either abusing or misusing or just the basic accidental; most pills, they'll look the same nowadays. So an older population they may not be able to see very well or read very well, they may just assume that they're taking the right medication, by chance, they end up taking the wrong medication. So they're more aware, more knowledgeable” (CPO 20) |
|  | Appealing to Females | “There was a lot more of probably 36 and up female... But it was more females that were interested in the pouches because of having their own children in the home or caring for an elderly” (CPO 01) |
|  | Instructions are Easy to Understand | “People say, "What? It's just that?" It's so simple and self-explanatory that people don't even believe that it's that simple” (CPO 34)  “It's simple enough to where a 16-year old can operate it to help an elderly parent to help their parent or to educate themselves from a presentation that we had or through a pamphlet that we give out” (CPO 01)  “I think what made it easy is the way that the instructions are worded is very user friendly” (CPO 22) |
|  | Staff Engagement with Intervention | “I have such a huge buy-in that I know that I could call any CPO member and say, "Hey, I need you to distribute 50 bags by the end of this month," and they would get it done, because we care that much” (CPO 02) |
|  | Increased Awareness Leads to Increased Intention to Use | “So once we started to give them the information, the tools that they needed, a lot of them were very interested. A lot of them were very receptive” (CPO 20)  “I think, as we've made people aware of them, it's made a lot of sense for people to use them. I've not had anybody that wasn't aware of the pouches because of our work, that asked for something like this” (CPO 18)  “It was the awareness that we wanted to put out there, that they didn't have to wait just from the Drug Take Back events, and they didn't have to flush them down toilets, or hoard them or whatever it was. It could actually […] something or be proactive” (CPO 20) |
|  | End User Engagement with Intervention | “Another positive thing is, that people are actually taking an account the severity of the opioid crisis, they're taking the initiative into great strides and saying, "We got to do something. Let's be proactive." They actually learn and they're wanting to know more information” (CPO 20)  “…to see the kind of community feedback that we get, and the acceptance that we get from the community with them, and the excitement in general” (CPO 22) |
|  | Engagement from Community Partnerships | “The people that I talked to, the people on the CPO that spoke with other people, they were all on board. I have a pretty good community that's trying to work towards things, making their community better. So, we're not getting a lot of pushback” (CPO 38) |
|  | IHDP Design is User Friendly | “But I think the ease and availability of the pouches is their best quality” (CPO 28)  “It's the size, the information is very accessible” (CPO 20)  “I think what was good is the fact that it is so easy. It's so user-friendly. It's black and white. It's not that hard to understand. You only have to use water. That's it. It's not like you have to have some kind of vinegar, or some kind of household cleaner, or something to help activate the charcoal, or whatever it is in there. So, it's easy” (CPO 22) |
|  | IHDP are Environmentally Friendly | “Yes, because we're saving the environment. If you think about the goods again, the pros, we're saving environment because they're not flushing the prescription drugs down the toilet” (CPO 26) |
|  | IHDP Increase Autonomy and Privacy of End-User | “I think easy to use and that you can use any time versus a take-back, or even we have several permanent drop boxes” (CPO 28)  “But at the end of the day, like I said, a lot of people are still fearful of going into the police department because of their immigrant status, or because lack of transportation. If we're able to actually go into the community and disperse these, they're easy to use” (CPO 42)  “I think privacy [what makes them suitable]. And then we had the option of doing the mail back or the pouch at home. And mainly we've been giving out disposal pouches…because they can see it's being dissolved and they can throw it away” (CPO 37) |
|  | Communities’ Familiarity with CPOs | “It's easy for me to just, we've been here for so long, and we're comfortable, and like I said, we work well with the community members, and also with the, in the schools, and attending our schools” (CPO 45) |
| Theme #2: The intervention is needed | Prevalence of Prescription Drug Misuse | “more people are talking about prescription drug issues within the elderly population because it is a concern” (CPO 37)  “Our suicide rate is also very high, which goes directly with misusing the prescription drugs and opioids” (CPO 19) |
|  | Limited Access to Other Disposal Methods | “And it was also a platform to be able to talk about the permanent drop box, which a lot of them may or may not have known because they live outside in the area, and transportation perhaps” (CPO 20) |
|  | Increased Prescribing | “So we prescribe at a higher rate than most other counties in Texas” (CPO 18)  “At one point about a year ago, there were 107 opioid prescriptions per every 100 residents. What that means is that there are people out in my community that have multiple prescriptions, two, three, or even more than that” (CPO 02) |
|  | Medication Hoarding | “One of the factors is, if residents in the rural area if they're unaware of the resources, they'll tend to hoard the medications whether they're expired or unused and save it for a later time, and that creates a problem because as you well know when you go to the doctor, you're more likely to get some medications. But if you don't have the money, or the insurance or even transportation, you may tend to hoard them for those specific reasons. And actually put yourself in a situation where you might be taking either too much or too little not making any good effect, and that can lead to other problems” (CPO 20) |
| Theme #3: CPO are creative with their Efforts | Bulk Distribution | “Actually the bulkheads, those are the people that I distributed the bulkheads to, to the inpatient treatment facilities” (CPO 27)  “So I was able to give them several hundred pouches that they then distributed straight back into our community, which I think was really effective” (CPO 02) |
|  | Strategic Distribution Efforts | “we don't usually just give a pouch to someone just because we need to give the pouches away, but we give them out when there's a need, so there's a greater likelihood that they're actually using the pouches for the correct reason versus just putting them aside somewhere in their house” (CPO 26) |
|  | Partnerships with Local Institutions and Community Liaisons | “We don't do a lot of our staff giving it to the end user. We try to work through a community partnerships that we already have” (CPO 18)  “We target the 12 sectors, which include law enforcement, the school, religious-based, community members, or business owners, and they attend our meetings every month” (CPO 20)  “We really focus a lot of our efforts on building capacity. What that means for us in real time is having these really solid working relationships with organizations in our community” (CPO 02) |
|  | Distribute at Local Community Venues | “That's kind of how we're using them now is trainings for opioid overdose and then education at fairs and handing them out to people who do take those prescriptions” (CPO 34)  “So we have several large events that we hold within the community that brings close to two to 300 families together” (CPO 01) |
|  | Creation of Resources | “We had built a website called …….. that we could point people towards” (CPO 18)  “We push it on our Facebook page as well, so we have a Facebook for the CPO” (CPO 38) |
|  | Education Promotion Efforts | “We do presentations where we distribute the pouches after, presentations for the pharmacists” (CPO 26)  “We did the presentation, the demonstration, how to use it and its benefits. I explained to them, a lot of you all are responsible for these patients, and disposing of their medications when they're discontinued from the doctor” (CPO 42) |
|  | Adapted Methods to Increase Reach | “I had one pharmacy that did a lot of mail order, so they were going to send there's with the patient's mail order as well for them to dispose of the other ones” (CPO 38)  “Another comment I wanted to add was that I believe one of our staff members had to type up some of this information in Spanish because we live in a predominantly Hispanic area where a lot of people do not speak English” (CPO 42) |
|  | CPO Modifications of IHDP | “we repackage them from the manufacturer's box into boxes of 70 when they were repacked in the clear bags” (CPO 37)  “…. the… area has a lot of Hispanics, we did add a sticker that had Spanish instructions on it. Now the postcard itself has Spanish on it but we added a sticker like a mail sticker, it's not very big we added it on, just stuck on the back to each one” (CPO 03) |
|  | Tailoring Messaging to Fit Audience | “Certainly if it's a parent presentation and I'm talking about current drug trends among teens, prescription drug abuse is a current drug trend and this makes it very suitable. And so I will target those groups for both my message and for the distribution” (CPO 03) |
| Theme #4: The intervention cost-related Factors | Availability of Other or Cheaper Alternatives | “They either already attended the DEA's event, which is the medication disposal program, or they just didn't have, so they wouldn't accept it [the IHDP]. That would be my only times and reasons when anyone else has said no to me, to where, no they didn't want any” (CPO 45)  “I think, because if somebody is going to pay to dispose of meds, I mean if I were to look at it from my perspective, it would be easier for me just to throw them in the trash versus paying X amount of money for pouch to dispose the meds properly” (CPO 01) |
|  | Cost Dictating Distribution | “So, if we don't have the funding or if we don't have the pouches, then that's going to take away the sustainability of trying to get those unused prescriptions out of the hands of the people in our community. Especially our elderly that may not have the transportation to get to a DEA take back, which is twice a year or to get to the Sheriff's Department” (CPO 38)  “We ourselves cannot purchase [IHDP], but my thought is it probably depends of the partnerships that we're going to be doing” (CPO 34) |
|  | Cost to End-User May Prevent Adoption of IHDP | “Yeah, if we had to pass that cost onto the end user, it would shrink to almost nothing” (CPO 18) |
|  | Cost to Third-Party May Prevent Adoption of IHDP | “The pharmacies were willing to, I think, continue to do it, just so long as they didn't have to buy the bags or pay for the bags” (CPO 38) |
|  | Acceptability of IHDP Cost | “And I think the price is reasonable for somebody that may only get 300 bucks a month or $600 a month on social security, but it's $5 to dispose of one month's worth of meds. I think it's a great investment” (CPO 01) |
| Theme #5: Addressing Structural/Process Factors that Inhibit Adoption of IHDP | Lack of Community Awareness Regarding IHDP (structural) | “And also, too, you're going to get people that go, "What? I don't understand what you're saying," or something like that. So, there is that kind of... I don't know. Pushback, a little bit” (CPO 22)  “And it's something that we also noticed that no one had ever heard of them before” (CPO 45) |
|  | Lack of Community Awareness Regarding the Risks of Prescription Drugs (structural) | “Most people don't know that it's that big of a problem. It seems like okay, it might be a problem in New York or somewhere else, but it's not a problem here. Opioid use is not a problem in our community. I think that's something we're seeing in our events is they don't really see it as a problem. They know it could be used, to be a problem, but I don't think they really understand it in our community” (CPO 34)  “They deny and are uneducated on the reason for why our community needs these” (CPO 19) |
|  | End User Uncomfortable with Disclosing Personal Information (process) | “Because people are always concerned about, "What do they want to know about me?" And I had to tell them, "Please let them know we're not trying to get any personal information on them." (CPO 38) |
|  | Increased Awareness Leading to Resource Strain (structural) | “Because once we started doing some bigger integrations with a big grocery store chains and a couple of big hospice providers, we were getting phone calls from other people wanting us to be able to do similar partnerships. And just having the pouches to be able to do that” (CPO 18) |
|  | Need for Active Marketing (process) | “the more we promote it, the initiative, the more receptive I think the community member” (  CPO 20)  “I think cost aside, the demand would not change because unfortunately, it's just people not being aware of the resources. They have to be told. If you're not in their face reminding them why it's important to properly dispose of medications, again, people are still wrapped up in the fact that they want to flush down the toilet or throw in the trashcan, and sometimes not even the correct way. They forget” (CPO 42) |
|  | Language Barriers (process) | “As it is here, there's the language barrier. For [the vender] to be able to provide leaflet information both in English and Spanish, it's a good thing because you target a whole bigger population, demographic area” (CPO 20)  “That it doesn't have it translated in Spanish on the back. I think that maybe people may take them, and then be like, "I really don't even understand this” (CPO 42) |
|  | Lack of information/misinformation in Rural Areas (structural) | “Absolutely, yes. And there's a very big difference on this like that big obstacle. So we do try to reach out outside the city limits because it seems that a lot of ... because its rural area, a lot of them are misinformed or don't even know about the resources available to them” (CPO 20)  “I would say that is an issue within our community being rural” (CPO 19) |
|  | Lack of Manpower (structural) | “Absolutely. We definitely need more volunteers. I'm just thinking we just need more volunteers who are willing to give up their time. Like I said, we had a few that were just always hands on. But it just takes manpower. I'm sure I'm not the only CPO who's facing that” (CPO 42)  “So, the more people that we get involved, the more time and individuals that we would need to make sure that we get those resources out to individuals as quickly as possible. So, it's going to take a lot more manpower, it's going to take, and more time” (CPO 38)  “So storage is always going to be an issue I think for most people but we managed very well” (CPO 03) |
|  | Covering Large and Far Service Areas (structural) | “We cover such a large geographic area. It can be an hour and 45 minute drive. To some of our partners” (CPO 18)  “Definitely one of the factors for us is distance of all our locations. […] County is a large county. It's very divided” (CPO 26)” |
|  | Younger Population Not as Engaged (process) | “Because a lot of times teens will just take a bag and be like, "Oh cool, what is this?" And go home and it just sits in the house” (CPO 19) |
|  | Difficulty in Tracking Distribution Efforts (process) | “As well as, it's just that part that I was saying with that, I wonder if they sent it? I wonder. That would be the only part if anything” (CPO 45)  “it would be very difficult for me to know who they're actually handing them out to distributing to” (CPO 20)  “Yeah. I don't have any way of tracking that at all. In good faith, we hope that they use it for the proper reasons, and they don't just throw it away, and they realize its value, and it's something that is good. You know, it needs to be used for the right reasons” (CPO 22) |
|  | Organizational Mishaps (process) | “That may be low ball-parking it because I know we had a staff member who left our agency a month after we received the patches, and we don't know what he distributed. That threw us off. We took that into consideration right away because we were like, oh, we don't know what he did. He didn't document that” (CPO 42) |
|  | Coverage of Counties not Catered to by Funded Organizations (process) | “Most of the people that we're talking to right now have been solely on board because they don't have a CPO there. And so, when someone's coming in and trying to help bring that change in their community, our community has been very receptive to us” (CPO 38)  “There are seven counties that touch our County within like 50 miles of the County. And 4 of those have CPOs, in the other 3, they're not” (CPO 37) |
|  | CPO Structural Change Due to State Funding (structural) | “Now that CPO it's kind of sad. It's kind of faded away because there's not a lot of involvement since there wasn't a funding source to it. And so our organization took it upon herself because we are in prevention education, and so a lot of the contacts I have, I'm able to disperse them. So the shipment came in like beginning of September, but my contract ended August 31^st^” (CPO 01) |
| Theme #6: Other organizations’ impact | Sharing of Monetary Burden | “My organization could say, "Hey, your CPO wants to do this, we could purchase the pouches and we can partner, and you guys can do the manpower and work on a Saturday" (CPO 01) |
|  | Interference from Outside Groups/Overlapping Services | “And then I did a presentation in October of this year and the same thing happened. They were like, "Oh yeah, [another CPO] had already came through and given us a thousand of these." We're like, "Oh, okay, well, what are you guys going to do with them?" They're like, "We're not sure yet” (CPO 18) |
|  | Collaboration with Other Similarly Oriented Groups | “And so we have partnered with another CPO that just started about two months ago, and I think they're in talks of getting the CCP contract for region one. And so just the barriers would be not having support from CPOs and also just manpower” (CPO 01) |
| Theme #7: CPOs interest to continue addressing implementation challenges | Targeting other Areas/Populations | “Interviewee: The expansion will probably be with the population.  Interviewer: Okay, not a geographic area but with the population.  Interviewee: Not a geographic area, yeah, but the expansion will be with the sectors of the community. For example, the veterans, the elderly community, we can expand to the nursing homes. Well, that goes with the elderly” (CPO 27) |
|  | Improving Structural Capacity | “Yeah. And if we expanded to those areas where we don't have CPOs, there would be no one there to provide education. So we will want to increase personnel to provide education in those communities as well. So then we could also talk about overdose prevention as well. The whole spectrum of prevention” (CPO 37) |
|  | Expand Partnership with Community Entities and Liaisons | “Because it's certainly going to take more manpower as we try to integrate more entities, inviting more schools, moving further out into the district. Even trying to get some of the local gyms to be a part, the local Y to be a part” (CPO 38) |
|  | Alternative Means of IHDP Distribution | “So this was the idea to set up a huge distribution of this pouches, but it would be more of a drive through, so that we could pull up and say, "How many would you like? Please fill out the survey right now." And then we'll take the card, drop it in a bucket or you can fill it out on your phone, and then you'll go” (CPO 01) |
|  | Develop Distribution Tracking Systems | “And then working with majority of your nursing homes, and I'm sure they have their own way of disposal, of course. I don't really know. I have to ask on how they dispose of their medications. So, yeah. That would be my approach, is to create a three question or two questions survey on top of what they're filling out for you guys, but for our records just to see if this is beneficial or not” (CPO 01) |
|  | Increase Focus on IHDP | “And I currently want to do the needs assessment plan or the strategic plan or the logic model. I include those Drug Take Back events, including the [IHDP] as a means to be able to facilitate the Drug Take Back events and other presentations. So that's already in place” (CPO 20) |
|  | Cost Predictions | “A case of 200 pouches is usually, what, anywhere from two to $3,000. We would of course need to factor that into our incentive budget, but it would just be a matter of us cutting back on something else where we can provide” (CPO 26) |
|  | Consequences of Expanding IHDP Distribution | “I think it could inhibit some of the progress we're making on some of our partnerships” (CPO 28)  “It would be a year round effort instead of us spending a few months a year, a couple of times a year on that” (CPO 18)  “I think cost would be the only negative. Cost in terms of staff and money” (CPO 28) |
|  | Effects of Expanding IHDP Distribution | “It would give us a chance to partner with more organizations in our community, to interact with more community members, educating them while creating awareness but not necessarily ... Yeah, more positives than anything else” (CPO 26)  “I mean, I really don't. Positive, I think it'd be a great way to... If we want to expand it, and reach out to other organizations to go in on it with us” (CPO 22)  “The expansion will definitely be... A positive effect will be less misuse. It will be less misuse, will definitely decrease the overdose rate, will definitely decrease the suicide rate. The community will be safer. The community will be more educated. Education is power. That will definitely be a positive effect, and the more we expand the better” (CPO 27) |
