# Supplementary material for: Evaluating the feasibility of implementing a prescription drug misuse prevention intervention in the community: a mixed methods study
Source: BMC Public Health. 2023 Apr 21;23:728. doi: 10.1186/s12889-023-15608-9 (PMC10120215; doi:10.1186/s12889-023-15608-9)
Supplement: Supplementary file 2 — Additional file 2. Feasibility Manuscript Final Coodebook. [file 12889_2023_15608_MOESM2_ESM.docx]

**Feasibility Final Codebook**

**ALL Transcripts were Arbitrated and Cleaned**

| **Theme** | | | | | |
| --- | --- | --- | --- | --- | --- |
| **#** | **Code** | **Description** | **# of Given Code per Group** | | |
|  |  |  | **High** | **Medium** | **Low** |

| **Theme #1: The intervention is desirable** | | | | | |
| --- | --- | --- | --- | --- | --- |
| **1** | Aligns with CPO Mission Goals | This code will be used any time the interviewers discuss how prescription drug misuse prevention, or medication disposal, or anything related to the IHDP is in concordance to their mission statements as a CPO. | 19 | 25 | 15 |
| **2** | Prior Experience with IHDP Intervention | This code refers to any discussion regarding whether the interviewed member and/or their CPO had experience with distributing and educating the end users on IHDP during an earlier period. | 4 | 1 | 6 |
| **3** | IHDP Being Supplemental | This code refers to any discussion regarding how the IHDP are an add-on to the CPO’s main activity instead of being the main focus at any event or venue. | 15 | 15 | 18 |
| **4** | Appealing to Schools | This code covers discussion regarding how IHDP make it easy for schools to dispose of their medication. | 2 | 2 | 3 |
| **5** | Appealing to Senior Citizens | This code is specific to any and all discussion about how IHDP are easier for senior citizens to use. Also any discussion of how senior citizens are appealed to IHDP more than other demographics. | 8 | 2 | 2 |
| **6** | Appealing to Females | This code refers to how IHDP were more appealing to or more accepted and adopted by females. | 5 | 1 | 1 |
| **7** | Instructions are Easy to Understand | This code refers to any and all discussion about how IHDP are easy to understand and do not require extensive explanation to people. | 3 | 8 | 4 |
| **8** | Staff Engagement with Intervention | This code will refer to any discussion pertaining to the staff or CPO members being engaged with the intervention and IHDP distribution. | 14 | 7 | 3 |
| **9** | Increased Awareness Leads to Increased Intention to Use | This code refers to any discussion regarding how once the CPOs increased awareness about the IHDP, they saw an increased acceptability of or demand for the IHDP. It is assumed that an increased interest in the IHDP indicates an intention to use the IHDP, if provided. | 14 | 7 | 4 |
| **10** | End User Engagement with Intervention | This code refers to end users approving of IHDP and voluntarily asking for them. | 12 | 9 | 7 |
| **11** | Engagement from Community Partnerships | This code refers to any interest expressed by community partnership institutions regarding the IHDP and their desire to be involved in the intervention. | 14 | 12 | 10 |
| **12** | IHDP Design is User Friendly | This code refers to any discussion regarding how the physical design and structure of the IHDP was user friendly to use, transport, instruct on, etc. | 6 | 8 | 5 |
| **13** | IHDP are Environmentally Friendly | This code covers any discussion concerning how IHDP are more environmentally friendly and that makes them appealing to the CPOs, end users, or the community in general. | 0 | 5 | 1 |
| **14** | IHDP Increase Autonomy and Privacy of End-User | This code encompasses all discussion regarding how the interviewed members explained that IHDP are popular because people can dispose of medication in their own homes, without assistance or oversight from other individuals. Also covers discussion regarding the increased level of privacy this affords the end users. This code DOES NOT encompass discussion about senior citizens. Any mention of senior citizens should be coded as “Appealing to Senior Citizens”. | 20 | 11 | 12 |
| **15** | Communities’ Familiarity with CPOs | This code refers to any discussion regarding how the CPO’s continued presence within their community has engendered trust/rapport within their community, so the end users are more likely to adopt the intervention. | 3 | 0 | 0 |

| **Theme #2: The intervention is needed** | | | | | |
| --- | --- | --- | --- | --- | --- |
| **16** | Prevalence of Prescription Drug Misuse | This code refers to any discussion involving the need for IHDP because of prescription drug misuse prevalence. Prescription drug misuse in this context can refer to people sharing medications, teenagers likely to take and sell medications, etc. It does not cover keeping medication after it is no longer needed. That will be covered under medication hoarding. | 7 | 7 | 2 |
| **17** | Limited Access to Other Disposal Methods | This code refers to any discussion regarding the need for IHDP as a result of limited access to other disposal options such as drug disposal drop boxes. | 9 | 3 | 3 |
| **18** | Increased Prescribing | This code refers to any discussion regarding high prescribing of medications by physicians. | 4 | 2 | 1 |
| **19** | Medication Hoarding | This refers to any discussion regarding the practice of keeping medication even after it is no longer needed for any reason. | 9 | 2 | 3 |

| **Theme #3: CPO are creative with their Efforts** | | | | | |
| --- | --- | --- | --- | --- | --- |
| **20** | Bulk Distribution | This code covers any discussion about how distributing the IHDP in bulk, rather than individually, has made things logistically easier on the CPO members. | 7 | 0 | 3 |
| **21** | Strategic Distribution Efforts | This code will be used for any discussion regarding how the CPOs have been “strategic” in their selection of partnerships, avenues of distribution, etc. in a way that has allowed them to minimize their own resource burden. | 13 | 18 | 11 |
| **22** | Partnerships with Local Institutions and Community Liaisons | This code refers to how the CPOs have partnered with local institutions (NOT other CPOs) such as pharmacies, student organizations, clinics, schools, etc. in order to further the distribution of IHDP. Also covers any discussion about how the CPO members have specific, individual contacts within the community that further their efforts to distribute IHDP. | 49 | 33 | 22 |
| **23** | Distribution at Local Community Venues | This code covers any discussion about the CPO members having attended community events and having the flexibility to distribute IHDP in a variety of avenues, even those not focused on prescription drug misuse prevention. | 28 | 16 | 20 |
| **24** | Creation of Resources | This code covers any discussion about novel things the CPOs did to encourage the adoption of IHDP among the end users. | 10 | 5 | 6 |
| **25** | Education Promotion Efforts | This code will refer to any efforts on the CPO’s part to increase educational promotion/trainings regarding the IHDP to the community that is independent of what the PREMIER Center has asked them to do (the handouts). | 30 | 21 | 11 |
| **26** | Adapted Methods to Increase Reach | This code refers to any discussion regarding how the CPOs had to adapt or change methods to reach a particular population. | 8 | 6 | 10 |
| **27** | CPO Modifications of IHDP | Code to discuss how the CPOs had to take an extra step (Ex: state didn’t print the cards in Spanish). | 4 | 3 | 4 |
| **28** | Tailoring Messaging to Fit Audience | This code refers to any discussion where the CPO members had to change the way they worded something in order to make their audience more receptive. This code is strictly limited to wording and dialogue. It does not cover their efforts to edit materials or activities to meet their audience. | 5 | 0 | 3 |

| **Theme #4: The intervention cost-related Factor** | | | | | |
| --- | --- | --- | --- | --- | --- |
| **29** | Availability of Other or Cheaper Alternatives | This code covers any discussion involving how the availability of other disposal options detracts from the adoption of IHDP – whether it’s because the other methods are cheaper or not. | 7 | 8 | 6 |
| **30** | Cost Dictating Distribution | This code covers any discussion involving how the cost of providing IHDP, either currently or in the future, can change the way the CPOs distribute them or even prevent them from future distributions. | 15 | 14 | 2 |
| **31** | Cost to End-User May Prevent Adoption of IHDP | This code refers to any discussion regarding how if there were a potential cost to the end user, however nominal, they probably would not use the IHDP. | 13 | 7 | 5 |
| **32** | Cost to Third-Party May Prevent Adoption of IHDP | This code refers to any discussion regarding how if there was a potential cost to a third-party (e.g., a nursing home, school, etc.), however nominal, they probably would not use the IHDP. | 4 | 4 | 2 |
| **33** | Acceptability of IHDP Cost | This code covers any discussion about how the cost of providing IHDP is reasonable given the value of them to the interviewee or the interviewee’s speculations on the end user/third party’s positive reaction to a potential cost. | 4 | 8 | 7 |

| **Theme #5: Addressing Structural/Process Factors that Inhibit Adoption of IHDP** | | | | | |
| --- | --- | --- | --- | --- | --- |
| **34** | Lack of Community Awareness Regarding IHDP (structural) | This code encompasses any discussion involving how the lack of awareness within the community has prevented people from recommending IHDP or using them regularly. | 15 | 7 | 4 |
| **35** | Lack of Community Awareness Regarding the Risks of Prescription Drugs (structural) | This code covers discussion about how members within the community are just unaware of the risks associated with prescription drugs and not properly disposing of them or properly storing them in an inaccessible location. | 4 | 8 | 2 |
| **36** | End User Uncomfortable with Disclosing Personal Information (process) | This code covers any discussion how end users may be hesitant to fill out the postcard attached to the IHDP for any reason. | 0 | 5 | 0 |
| **37** | Increased Awareness Leading to Resource Strain (structural) | This code refers to how as the CPOs increased their distribution and community awareness of the IHDP; the increased demand became difficult to keep up with. | 2 | 0 | 0 |
| **38** | Need for Active Marketing (process) | This code refers to any discussion regarding how the public had to be continually reminded or pushed to use the IHDP because otherwise they would “forget” about them. | 3 | 7 | 9 |
| **39** | Language Barriers (process) | This code refers to any discussion about how there was a language barrier, either with the members of the CPO and their interaction with people or with the materials distributed not being in another language. | 3 | 2 | 4 |
| **40** | Lack of Information/Misinformation in Rural Areas (structural) | This code refers to any discussion regarding how the rurality of the locations a CPO served impacted distribution of the IHDP. | 6 | 2 | 0 |
| **41** | Lack of Manpower (structural) | This code refers to how the CPOs may simply not have the infrastructure necessary to house the volume of IHDP they are either currently receiving or may receive in the future. | 9 | 5 | 16 |
| **42** | Covering Large and Far Service Areas (structural) | This code refers to any particular features of the area the CPO serves that makes it difficult to distribute the intervention. | 4 | 4 | 3 |
| **43** | Younger Population Not as Engaged (process) | This code refers to discussion regarding how younger populations aren’t as interested or seem less likely to participate in the intervention. | 4 | 2 | 1 |
| **44** | Difficulty in Tracking Distribution Efforts (process) | This code refers to any discussion pertaining to CPO members explaining how they had difficulty in tracking their distribution efforts. | 16 | 10 | 13 |
| **45** | Organizational Mishaps (process) | This code refers to any mishap on the part of the CPOs in terms of not receiving the IHDP in time, not organizing in a timely manner, etc. that prevented dissemination of the intervention. | 0 | 0 | 10 |
| **46** | Coverage of Counties not Catered to by Funded Organizations (process) | This code ma refers to any discussion regarding how the CPOs are covering more than just their area because there are certain counties that are not covered by any funded organization. | 5 | 1 | 0 |
| **47** | CPO Structural Change Due to State Funding (structural) | This code encompasses any discussion about how funding status changed depending on state funding requirements. | 4 | 0 | 15 |

| **Theme #6: Other organizations’ impact** | | | | | |
| --- | --- | --- | --- | --- | --- |
| **48** | Sharing of Monetary Burden | This code refers to any discussion regarding how the institutions that the CPOs have been providing IHDP for could/have already helped by sharing the financial burden associated with the intervention – whether that be for the educational materials or the IHDP. | 4 | 3 | 4 |
| **49** | Interference from Outside Groups/Overlapping Services | This code refers to any discussion regarding how other CPOs have given people within their area the IHDP, but they did it with no plan to educate them on how to use them. | 4 | 0 | 0 |
| **50** | Collaboration with Other Similarly Oriented Groups | This code refers to how partnering with other CPOs has made distribution easier on any individual CPO. | 7 | 1 | 6 |

| **Theme #7: CPOs interest to continue addressing implementation challenges** | | | | | |
| --- | --- | --- | --- | --- | --- |
| **51** | Targeting other Areas/Populations | This code refers to any discussion regarding the CPOs’ plans to include and cover other populations or geographic areas besides the ones they are currently serving. | 4 | 0 | 2 |
| **52** | Improve Structural Capacity | This code refers to any discussion regarding the CPOs’ plans to provide the needed infrastructure necessary to house the volume of IHDP they may receive in the future, or/and personnel. | 1 | 1 | 0 |
| **53** | Expand Partnership with Community Entities and Liaisons | This code refers to any discussion about the CPOs’ plans to engage in partnerships with local institutions (NOT other CPOs) in order to further the distribution of IHDP, in addition to the entities they are currently working with. | 3 | 3 | 1 |
| **54** | Alternative Means of IHDP Distribution | This code refers to any discussion about the CPOs’ plans to utilize other distribution methods besides the ones currently used. | 0 | 1 | 1 |
| **55** | Develop Distribution Tracking Systems | This code refers to any discussion about the CPOs’ plans to create a tracking system for any activities related to IHDP distribution. | 0 | 0 | 2 |
| **56** | Increase Focus on IHDP | This code refers to any discussion regarding the CPOs stating that in the future they will have more of a focus on distributing the IHDP and delivering the education associated with it. | 1 | 0 | 0 |
| **57** | Cost Predictions | This code will be used for any discussion regarding the cost in expanding their current distribution area or efforts. | 3 | 1 | 1 |
| **58** | Consequences of Expanding IHDP Distribution | This code will be used for any discussion regarding what the CPOs would have to sacrifice, in terms of time or focus on, in order to make sure that the IHDP are being distributed as well as they could be. This code is strictly limited to only the negative impact of expanding IHDP distribution. | 4 | 1 | 7 |
| **59** | Effects of Expanding IHDP Distribution | This code refers to the positive effects of expanding IHDP Distribution. | 5 | 5 | 7 |
